# Effects of Restricted Blood Flow Interval Training on Lower Extremity Muscles and Motor Function in Stroke Patients

**DOI:** 10.1002/brb3.70683

**Published:** 2025-07-10

**Authors:** Yongxiang Li, Yali Liu, Jiangrong Xiong

**Affiliations:** ^1^ Department of Rehabilitation Medicine, Center for Rehabilitation Medicine, the Fourth Affiliated Hospital of School of Medicine, and International School of Medicine, International Institutes of Medicine Zhejiang University Yiwu China; ^2^ Department of Infectious Diseases, Center for Infectious Diseases, the Fourth Affiliated Hospital of School of Medicine, and International School of Medicine, International Institutes of Medicine Zhejiang University Yiwu China; ^3^ Department of Rehabilitation Medicine The First Affiliated Hospital of Zhejiang Chinese Medical University (Zhejiang Provincial Hospital of Chinese Medicine) Hangzhou Zhejiang China; ^4^ Department of Rehabilitation, Xinhua Hospital, School of Medicine Shanghai Jiao Tong University Shanghai China

**Keywords:** blood flow restriction, gastrocnemius pinna angle, rectus femoris cross‐sectional area, rectus femoris muscle thickness, stroke

## Abstract

**Objective:**

To examine how limitations in blood circulation impact the training of stroke individuals.

**Methods:**

Between March 2022 and March 2023, a total of 34 individuals receiving treatment at the Fourth Affiliated Hospital of the School of Medicine, Zhejiang University, specifically within the Department of Rehabilitation Medicine, were chosen as participants. They were then assigned to experimental groups using a random number approach, with 17 individuals in each group, while also including a control group. The test group received BFR combined with cycle ergometers, while the control group performed a cycle ergometers regularly. Ultrasonography was employed to assess the size and thickness (RFT) of the rectus femoris (RFSTA) in patients both prior to and following training, as well as to evaluate the angle of the gastrocnemius pinna. Additionally, each patient completed a 30‐s sit‐to‐stand test, received results from a stretch test, and underwent the Fugl‐Meyer assessment for the lower extremities.

**Results:**

The muscles of RFT, RFTSA, and gastrocnemius pinna angle did not change significantly before and after in the control group. However, these values increased markedly in the experimental group. In addition, the FMA value recorded in the test group notably surpassed that of the control group. After all, walking speed, frequency, length and overall mobility will increase after training, but you will find it more important.

**Conclusion:**

BFR can promote rehabilitation functional, relieve stress, ensure safety, improve training effects and have high value clinical uses.

## Graphical abstract

BFR can promote the functional Rehabilitation of stroke patients and strengthen their motor ability without high ‐intensity and ensure safety, which has high clinical application value.

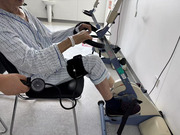

## 1 Introduction

Stroke is characterized by a sudden onset of progressive cerebral ischemia or hemorrhage, presenting with frequent clinical features, lesions, recurrence, and high mortality rates. It stands as the leading cause of death globally, surpassed only by heart disease and ischemic conditions (Feigin et al. 2017). In the past few years, the occurrence of stroke has risen significantly, and research has increasingly shown that it can affect younger individuals (Mozaffarian et al. 2016). According to statistics, the global frequency reached 1.0% –3.0% in 2020, with a monthly mortality rate of 3.0%–5.0% (Feske 2021; Broderick et al. 2021; Cordonnier et al. 2018). The principles of clinical treatment are currently diagnosis, early treatment, early rehabilitation, and prevention of recurrence. Improving cerebral ischemic blood circulation promotes neurological function and reduces mortality in stroke patients (Sarfo et al. 2018; Kuriakose and Xiao 2020). However, the risk of disease after stroke treatment is a hot issue and should be effectively addressed in clinical practice (Ginsberg 2018; Dusenbury and Alexandrov 2020). Given the high disability rate, rehabilitation training is often required after treatment. Restoring the patient's ability to control their movements autonomously is crucial for achieving independence and facilitating functional recovery (Dusenbury and Alexandrov 2020). However, the main problem is that traditional high‐intensity training may cause complications and side effects during the patient's rehabilitation process and the inability to achieve the expected impact of rehabilitation training to reduce the intensity of the training (Tegtbur et al. 2020). Consequently, it is essential to identify a secure and efficient training regimen that is most appropriate for individuals recovering from a stroke.

Blood flow restriction training (BFR) represents an emerging technique for rehabilitation that primarily employs elastic wrapping devices (including items like elastic bands, tourniquets, and cuffs) to exert pressure, thereby restricting the return of blood from the distal veins to the body. This method is paired with lower exercise intensities to promote muscle development and enhance exercise adaptability (Lorenz et al. 2021). Studies have shown that BFR can reduce the atrophy of muscle fibers and increase muscle volume (Ferraz et al. 2018). It is perfect for stroke patients to recover. However, no clinical studies have confirmed the accurate effect of BFR on stroke.

Therefore, BFR has analyzed the impact of new stroke reports on stroke recovery and provides a more reliable guarantee for improving predictions.

## 2 Clinical Data and Methods

### 2.1 Case Source

Between March 2022 and March 2023, a total of 34 individuals from the Department of Rehabilitation Medicine were chosen for assessment and subsequently divided into experimental groups, with the selection process based on random numbers (*n* = 17). The group undergoing testing received BFR therapy in conjunction with cycle ergometers, while the control group utilized cycle ergometers alone. The research was approved by the Ethics Committee, and all participants provided informed consent. The determination of sample size was conducted through a preliminary power analysis conducted using G*Power software (version 3.1). The analysis aimed to establish a robust framework for the study, with an anticipated effect size set at 0.8, a significance threshold of 0.05, and a power of 0.8. It was necessary to have at least 15 participants in each group. To mitigate the impact of possible dropouts, we opted to include 17 participants in both groups.

### 2.2 Selection Criteria

Included standards: (1) Comply with diagnostic criteria of stroke (Ziai and Carhuapoma 2018) and confirm by CT or magnetic. (2) The onset and initial progress of the disease should last less than 6 months. (3) Seated balance level 2 (Van Criekinge et al. 2019). (4) The patient signed the consent form. Exclusion criteria: (1) Significant cognitive deficits and a lack of willingness to engage in rehabilitation evaluation and therapy. (2) Significant cardiovascular conditions, as opposed to managed hypertension (with blood pressure exceeding 160/100 mmHg), along with peripheral arterial issues. (3) Have lower extremity venous thrombosis. (4) Complex peripheral neuropathy. (5) Patients receiving anticoagulants and dual antiplatelet treatments. (6) Patients suffering from other serious organs or systemic diseases.

### 2.3 Training Methods

Experimental group: General training on a cycle ergometers under rehabilitation physician's instructions and support. Before training, place the patient in a sitting position before training and tie the inflatable cuff 10 cm wide to the thigh. Press the initial cuff on the blood pressure cramp for 30 s, relax for 30 s, and followed by an additional pressure of 20 mmHg on top of the previous systolic pressure, then relax for 10 s. Repeat until inflation reaches 200 mmHg and scanned femoral arterial occlusion with ultrasound. at which point the pressure is the pressure of arterial occlusion (AOP). Bend the proximal lower limbs near the groin and determine the cuff pressure to be 40%–80% AOP. Each exercise lasts for 2 min, rest for 1 min, and lasts for 10 min. The training time is 5 days a week, 2 times a day, for a total of 3 weeks. Keep the pressure between training and between releasing the pressure after the last set was over. Warm up before training and relax after training. Control group: These methods are the same as above, but use 10% AOP cuff pressure. The 10% AOP (arterial occlusion pressure) in the control group was selected to simulate the physical sensation of cuff application without inducing substantial vascular restriction.

### 2.4 Outcome Measures

Ultrasonography was employed both prior to and following the training sessions to assess the size and thickness (RFT) of the rectus femoris (RFSTA) in patients, as well as to evaluate the angle of the gastrocnemius pinna. For the 30‐s sit‐stand test, the individual was positioned in a chair, ensuring that their feet were aligned with shoulder width and their arms were placed in front of their chest. Write down how often the patient leaves the chair within 30 s. Stretch test: A leveled “yardstick” is fixed to very high walls. It keeps your feet on the same floor. The participants were invited to strip the fist and reach out, and then the third metacarpal position was recorded. Participants were asked to expand as much as possible without losing balance or activity and recorded the location of the third metacarpal. The lower extremity of Fugl‐Meyer (FMA) was evaluated (Gladstone et al. 2002): 34 points, the better the function of patients with lower extremity. In order to reduce assessment bias, evaluations of outcomes were carried out by a qualified rehabilitation expert who remained unaware of the group assignments during the entire study.

### 2.5 Statistical Analysis

The gathered data underwent statistical evaluation using the statistical analysis software SPSS 23.0. Count data are expressed as percentages of usage (%), and chi‐squared tests were employed for comparative analysis. For measurement data, the mean ± standard deviation was utilized. For group comparisons, independent sample *t*‐tests were used, and paired *t*‐tests were conducted before and after the training sessions. *p* < 0.05 was statistically significant.

## 3 Results

### 3.1 Patient Clinical Data

No statistically meaningful difference was observed between with the clinical data of the two patient groups, and the results were comparable (Table 1).

**TABLE 1:** Patient clinical data.

	Control group (*n* = 17)	Experimental group (*n* = 17)	*p*
Gender			0.5
Male	88.24% (15)	82.36% (14)	
Female	11.76% (2)	17.64% (3)	
Age	64.41 ± 12.90	59.59 ± 8.95	0.173
Height (cm)	167.88 ± 5.48	170.12 ± 4.48	0.201
Weight (kg)	75.29 ± 16.79	74.94 ± 8.19	0.939
BMI (kg/m^2^)	26.51 ± 4.61	25.95 ± 2.09	0.651
Stroke type			0.486
Cerebral infarction	35.29% (6)	47.06% (8)	
Cerebral hemorrhage	64.71% (11)	52.94% (9)	
Course of disease (days)	41.53 ± 33.50	47.06 ± 42.26	0.675
Affected side			0.5
Left	88.24% (15)	82.36% (14)	
Right	11.76% (2)	17.64% (3)	
AOP (mmHg)	180.06 ± 13.86	183.94 ± 10.45	0.364

### 3.2 RFT Versus RFSTA

**FIGURE 1:**
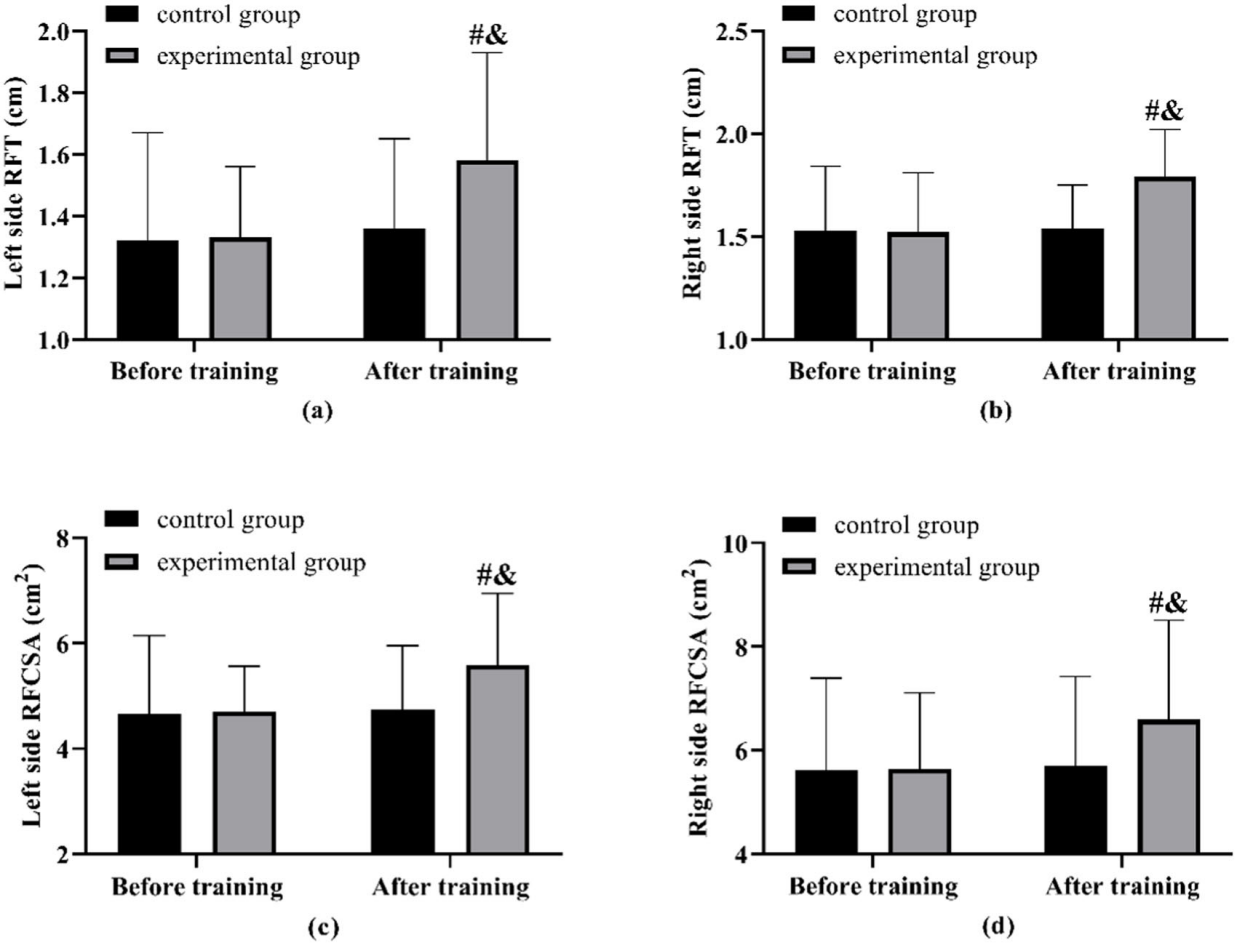
**RFT versus RFSTA**. (a) Comparison of left side RFT before and after training in both groups. (b) Comparison of right side RFT before and after training in both groups. (c) Comparison of left side RFSTA before and after training in both groups. (d) Comparison of right side RFSTA before and after training in both groups. Note: compared with before training ^#^(*p* < 0.05), compared with the control group ^&^(*p* < 0.05).

Prior to the training, the two groups exhibited no notable differences in RFT and RFTA. Following the training, RFT and RFSTA were shown no changes in the control group. In contrast, the experimental group demonstrated increased values for both measurements on the left and right sides compared to their pre‐training levels, as well as higher values than those observed in the control group (Figure 1).

### 3.3 Gastrocnemius Pinna Angle Comparisons

**FIGURE 2:**
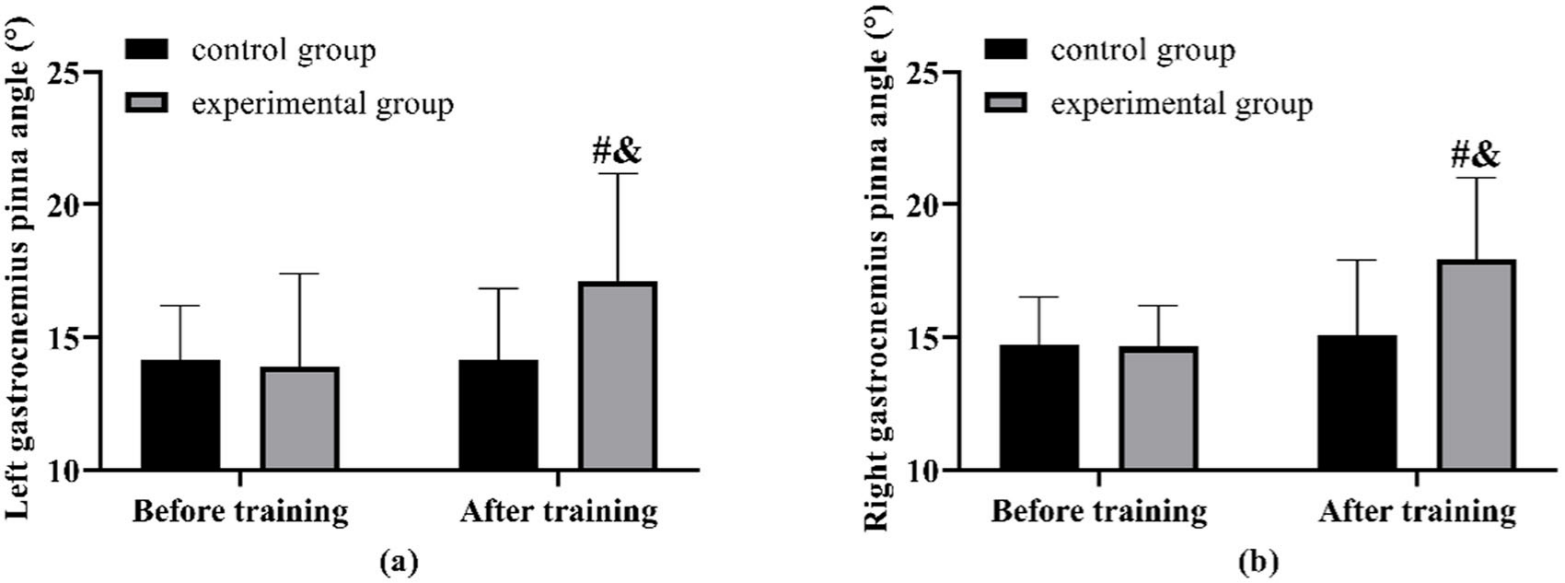
**Comparison of gastrocnemius pinna angles**. (a) Comparison of left gastrocnemius pinna angle before and after training in both groups. (b) Comparison of right gastrocnemius pinna angle before and after training in both groups. Note: compared with before training ^#^(*p* < 0.05), compared with the control group ^&^(*p* < 0.05).

The gastrocnemius pinna angle showed no notable difference between the two groups prior to the training. Following the training period, the test group exhibited an increase in the gastrocnemius pinna angle. This increase was greater than that observed in the control group, which, in contrast, did not show any change when compared to its measurements before training (Figure 2).

### 3.4 Comparison of Lower Limb Muscle Strength and Function

**FIGURE 3:**
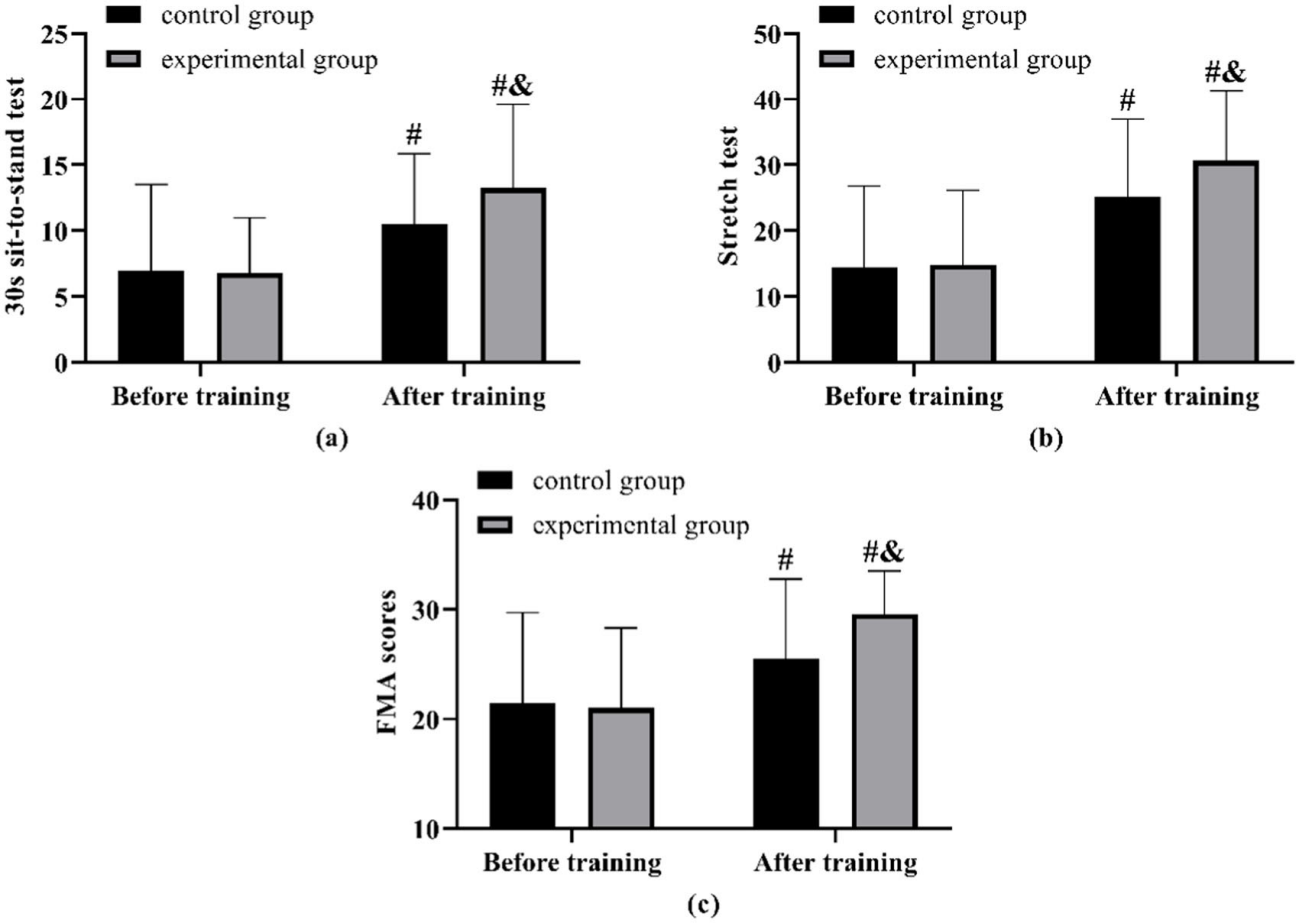
**Comparison of lower limb muscle strength and function**. (a) Comparison of results of the 30s sit‐to‐stand test before and after training. (b) Comparison of stretch test results before and after training. (c) Comparison of FMA scores before and after training. Note: compared with before training ^#^(*p* < 0.05), compared with the control group ^&^(*p* < 0.05)).

No notable differences were observed in the sit‐to‐stand test, stretch test, and FMA outcomes between the two groups prior to the training. Following the training, improvements were noted in the results of the 30‐s sit‐to‐stand test, stretch test, and FMA scores for both groups, with the experimental group demonstrating superior results in each assessment compared to the control group (Figure 3).

## 4 Discussion

The application of BFR training is a potentially effective approach in the field of neurorehabilitation. It enables low‐load resistance exercise to produce similar physiological benefits to high‐intensity training, which is particularly advantageous for patients with limited physical capacity after stroke (Kilgas et al. 2019). The clinical rationale lies in minimizing mechanical stress on joints and cardiovascular systems while still promoting muscular and neurological adaptations (Rolff et al. 2020). The processes that explain the effects of BFR are still not completely comprehended. It is thought that BFR generates heightened metabolic stress by establishing a low‐oxygen environment, which in turn encourages the recruitment of muscle fibers and triggers intracellular signaling pathways linked to hypertrophy (Lipker et al. 2019). These include elevations in lactate, growth hormone secretion, and enhanced muscle protein synthesis even under low mechanical loading (Bowman et al. 2019). Several studies have confirmed that BFR leads to measurable improvements in muscle volume, strength, and functional capacity across clinical populations (Saatmann et al. 2021; Pearson and Hussain 2015).

In the context of stroke rehabilitation, BFR shows particular promise. Recent evidence has demonstrated that BFR training can facilitate motor recovery, improve muscle morphology, and enhance neuromuscular coordination through both peripheral and central adaptations (Baker et al. 2020; Rodrigo‐Mallorca et al. 2021). A scoping review conducted by Cummings and Madhavan emphasizes that the modulation of blood flow can have a beneficial impact on motor outcomes and neurophysiological processes in stroke patients. In a similar vein, a randomized controlled trial by Ahmed et al. (Ahmed et al. 2024) found that BFR led to notably enhanced improvements in muscle strength of the lower limbs and motor function when compared to conventional resistance training among individuals who have experienced ischemic strokes.

Our current findings are consistent with these previous reports. This research revealed that the BFR group showed notable enhancements in the thickness of the rectus femoris muscle (RFT), the cross‐sectional area (RFSTA), and the pennation angle of the gastrocnemius, compared to the control group. Additionally, scores from functional assessments such as the 30‐s sit‐to‐stand test, the stretch test, and the lower extremity Fugl‐Meyer Assessment (FMA) exhibited greater improvements in the BFR group. These results indicate that BFR could contribute to promoting both muscular and functional recovery among individuals who have experienced a stroke.

Additionally, BFR has been shown to support tendon remodeling and musculoskeletal adaptation (Robertson et al. 2023; Centner et al. 2019). It promotes vascular responses and skeletal muscle reconditioning, which are critical in post‐stroke populations where disuse atrophy and poor circulation are prevalent. At the cellular level, BFR may upregulate growth‐related signaling pathways such as mTOR, contributing to increased muscle mass and contractile function (Minniti et al. 2020; Hughes et al. 2018). These findings support the hypothesis that BFR has both localized and systemic benefits in rehabilitation. The safety and tolerability of BFR are also supported by growing clinical evidence. While theoretical risks include delayed‐onset muscle soreness (DOMS), venous congestion, or vascular complications, studies in stroke and elderly populations suggest that BFR is generally well‐tolerated with appropriate monitoring (Cummings and Madhavan 2024). In our study, no serious adverse events were reported. Minor discomfort during cuff inflation or transient muscle soreness resolved without intervention. These findings reinforce the feasibility of implementing BFR as a routine adjunct in stroke rehabilitation.

Finally, BFR is highly compatible with clinical workflows. It can be performed with minimal equipment, adapted to inpatient or outpatient settings, and integrated alongside conventional physical therapy. Its ability to deliver effective rehabilitation outcomes while reducing training load makes it a valuable tool for clinicians aiming to restore motor function in vulnerable populations (Ahmed et al. 2024). However, this study has several limitations. Initially, the intervention lasted just 3 weeks, a timeframe that is inadequate for assessing the long‐term impacts or sustainability of BFR training in the context of stroke rehabilitation. Future studies should incorporate extended follow‐up periods to assess the persistence of functional improvements and the safety of prolonged BFR use. Additionally, several confounding factors, such as nutritional status, baseline muscle strength, and daily activity levels, were not fully controlled and may have introduced potential bias. Addressing these limitations in future research will help to provide more robust evidence regarding the clinical value of BFR. Furthermore, although we recognized the potential influence of baseline muscle strength, nutritional status, and overall physical activity levels, it was challenging to standardize these variables across all participants. Stroke patients often present with heterogeneous health conditions and varying degrees of physical limitations, making strict control or unification of these factors impractical in clinical settings. Nonetheless, all participants were enrolled under the same inclusion criteria and received comparable instructions to minimize variability as much as possible.

## 5 Conclusion

BFR has the potential to enhance the functional recovery of individuals who have experienced a stroke while also improving their motor skills. This is achieved by minimizing the physical strain and prioritizing safety, making it highly valuable in clinical settings.

## Author Contributions

**Yongxiang Li**: methodology, writing–original draft. **Yali Liu**: writing–review and editing. **Jiangrong Xiong**: formal analysis, data curation.

## Conflicts of Interest

The authors declare no competing interests.

## Ethics Statement

All procedures involving human participants followed ethical standards set by institutional and/or national research committees.

## Informed Consent

Informed consent was obtained from all participants or their legal guardians.

## Peer Review

The peer review history for this article is available at https://publons.com/publon/10.1002/brb3.70683.

## Data Availability

The datasets are available from the corresponding author upon request.
