# Correction to “Effects of Restricted Blood Flow Interval Training on Lower Extremity Muscles and Motor Function in Stroke Patients”

**DOI:** 10.1002/brb3.70802

**Published:** 2025-09-25

**Authors:** Jianrong Xiong

Li, Y., Y. Liu, and J. Xiong. 2025. “Effects of Restricted Blood Flow Interval Training on Lower Extremity Muscles and Motor Function in Stroke Patients.” *Brain and Behavior* 15, no. 7: e70683.

**[The corresponding author's name was misspelled as “Jiangrong Xiong.” The correct spelling is “Jianrong Xiong.”**

The first author's affiliation “Department of Rehabilitation Medicine, Center for Rehabilitation Medicine, the Fourth Affiliated Hospital of School of Medicine, and International School of Medicine, International Institutes of Medicine, Zhejiang University, Yiwu, China” was incorrect because we have just been informed by the relevant department at our hospital that the “Center for Rehabilitation Medicine” is not set up at our institution now. The first author's correct affiliation is “Department of Rehabilitation Medicine, the Fourth Affiliated Hospital of School of Medicine, and International School of Medicine, International Institutes of Medicine, Zhejiang University, Yiwu, China.”

The second author's affiliation “Department of Infectious Diseases, Center for Infectious Diseases, the Fourth Affiliated Hospital of School of Medicine, and International School of Medicine, International Institutes of Medicine, Zhejiang University, Yiwu, China” was incorrect because the “Center for Infectious Diseases” is wrong. The second author's correct affiliation is “Department of Infectious Diseases, the Fourth Affiliated Hospital of School of Medicine, and International School of Medicine, International Institutes of Medicine, Zhejiang University, Yiwu, China.”

We apologize for this error.

